# Development of an Artificial Intelligence Model for Analyzing the Relationship between Imaging Features and Glucocorticoid Sensitivity in Idiopathic Interstitial Pneumonia

**DOI:** 10.3390/ijerph192013099

**Published:** 2022-10-12

**Authors:** Ling Jiang, Meijiao Li, Han Jiang, Liyuan Tao, Wei Yang, Huishu Yuan, Bei He

**Affiliations:** 1 Department of Respiratory and Critical Care Medicine, Peking University Third Hospital, Beijing 100191, China; 2 Department of Radiology, Peking University Third Hospital, Beijing 100191, China; 3 OpenBayes (Tianjin) IT Co., Ltd., Beijing 100027, China; 4 Research Center of Clinical Epidemiology, Peking University Third Hospital, Beijing 100191, China

**Keywords:** artificial intelligence, idiopathic interstitial pneumonia, imaging features, glucocorticoid sensitivity

## Abstract

High-resolution CT (HRCT) imaging features of idiopathic interstitial pneumonia (IIP) patients are related to glucocorticoid sensitivity. This study aimed to develop an artificial intelligence model to assess glucocorticoid efficacy according to the HRCT imaging features of IIP. The medical records and chest HRCT images of 150 patients with IIP were analyzed retrospectively. The U-net framework was used to create a model for recognizing different imaging features, including ground glass opacities, reticulations, honeycombing, and consolidations. Then, the area ratio of those imaging features was calculated automatically. Forty-five patients were treated with glucocorticoids, and according to the drug efficacy, they were divided into a glucocorticoid-sensitive group and a glucocorticoid-insensitive group. Models assessing the correlation between imaging features and glucocorticoid sensitivity were established using the k-nearest neighbor (KNN) algorithm. The total accuracy (ACC) and mean intersection over union (mIoU) of the U-net model were 0.9755 and 0.4296, respectively. Out of the 45 patients treated with glucocorticoids, 34 and 11 were placed in the glucocorticoid-sensitive and glucocorticoid-insensitive groups, respectively. The KNN-based model had an accuracy of 0.82. An artificial intelligence model was successfully developed for recognizing different imaging features of IIP and a preliminary model for assessing the correlation between imaging features and glucocorticoid sensitivity in IIP patients was established.

## 1. Introduction

Idiopathic interstitial pneumonia (IIP) is a pulmonary interstitial disease of unknown etiology that is difficult to diagnose and treat. A combination of clinical features, imaging examinations, and pathology are needed to diagnose the disease. The pathology is difficult to obtain. If the patient is not treated in a timely manner, the prognosis is poor. Idiopathic pulmonary fibrosis (IPF) is a common type of IIP that can be diagnosed directly without pathological examination if its typical imaging features are seen in HRCT. However, for IPF with atypical CT manifestations and other non-IPF patients, few treatment methods are available, and only some can be effectively treated with glucocorticoid therapy. If IIP patients sensitive to glucocorticoid therapy can be identified early, it should be started as soon as possible to improve the prognosis. Previous studies [1,2,3,4,5,6,7] have shown that the HRCT imaging characteristics of IIP patients are related to glucocorticoid efficacy. In HRCT, ground glass opacities are mostly related to the active inflammation of the alveoli and alveolar interstitium, which is sensitive to glucocorticoid therapy. In contrast, reticulations and honeycombing are mostly related to alveolar septal fibrosis, which is not sensitive to glucocorticoid therapy, and indicate a poor prognosis. A patient diagnosed with IIP can present with multiple imaging features at the same time, and their area percentages are related to their sensitivity to glucocorticoid therapy: the greater the area percentage of reticulations and honeycombing, the lower the sensitivity to glucocorticoids. The area proportions of imaging features will not be reported in reality. Previous studies have shown that the proportion of different imaging features obtained through manual calculation is related to glucocorticoid sensitivity [2,3]. In recent years, artificial intelligence methods have been widely used in the fields of lung imaging data and diagnosis [8,9,10,11,12,13]. The development of artificial intelligence technology makes it possible to automatically identify imaging features and calculate their proportions. Based on the actual glucocorticoid sensitivity grouping, a hypothesis can be proposed to establish an artificial intelligence model to judge the glucocorticoid sensitivity through HRCT. Due to the low incidence of IIP, no stable artificial intelligence model has been established, particularly one that could assess the correlation between imaging data and glucocorticoid sensitivity. The goal of this study is to establish an HRCT imaging feature recognition model for IIP and a model for preliminarily assessing the correlation between imaging features and glucocorticoid sensitivity with artificial intelligence algorithms.

## 2. Materials and Methods

### 2.1. Objects

**Figure 1:**
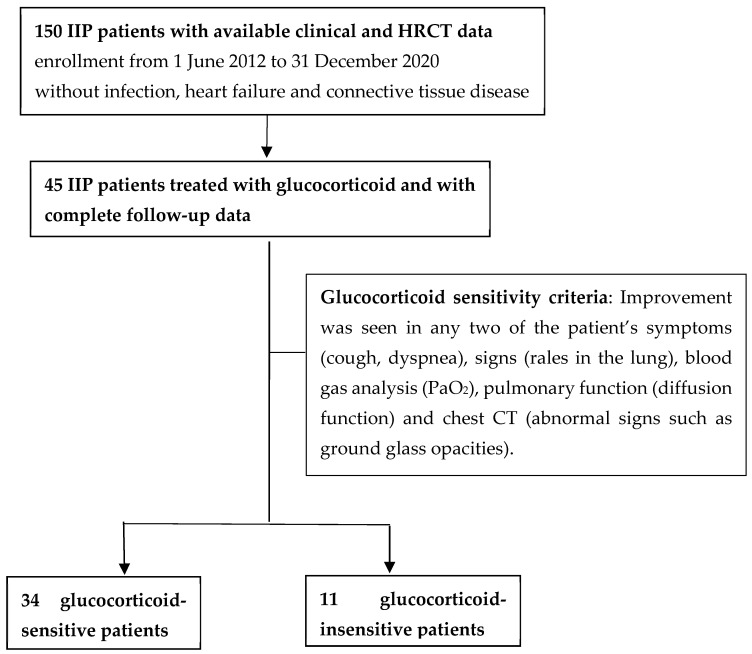
Glucocorticoid-sensitivity grouping process.

A retrospective case–control study was conducted to collect 150 IIP inpatients treated in the Department of Respiratory and Critical Care Medicine of the Peking University Third Hospital from 1 June 2012 to 31 December 2020. The inclusion criteria included the following: IIP diagnostic criteria as defined by the international multidisciplinary consensus on IIP classification published by the American Thoracic Society (ATS)/European Respiratory Society (ERS) in 2013; a diagnosis of IIP by a clinician, pathologist, or radiologists, including the following pathological types: idiopathic pulmonary fibrosis (IPF), nonspecific interstitial pneumonia, cryptogenic organic pneumonia, acute interstitial pneumonia, respiratory bronchiolitis, interstitial lung disease (ILD), and desquamation interstitial pneumonia; and complete clinical and imaging data. The exclusion criteria were as follows: incomplete clinical or imaging data, and complications of infection, heart failure, or connective tissue disease. The medical records of 150 patients with IIP were reviewed. Forty-five patients were treated with glucocorticoids and had complete follow-up data. According to glucocorticoid efficacy, these 45 patients were divided into a glucocorticoid-sensitive group (34 patients) and a glucocorticoid-insensitive group (11 patients). The glucocorticoid-sensitivity grouping process is presented in Figure 1.The glucocorticoid dosage was 0.5–1 mg/kg/d. The criteria for judging glucocorticoid efficacy were as follows: if an improvement was seen in any two of the patient’s symptoms (cough and dyspnea), signs (rales in the lung), blood gas analysis (PaO_2_), pulmonary function (diffusion function), and chest CT (abnormal signs such as ground glass opacities), the patient was placed in the glucocorticoid-sensitive group; otherwise, the patient was placed in the glucocorticoid-insensitive group. The criteria for the improvement of each index are as follows: symptoms: the complete absence of cough or a reduction in its intensity and frequency and increased endurance while performing daily activities; signs: reduced or absent; PaO_2_: increased by ≥4 mmHg; pulmonary function: diffusion capacity of lung for carbon dioxide (DLCO) increased by ≥10%; and chest CT: abnormal signs reduced or absent abnormal signs.

### 2.2. Imaging Data Collection and Manual Labeling

Chest CT scans were performed on a Siemens Somatom Definition Flash CT machine or a GE Discovery CT750HD. The scanning range was from the apex to the bottom of the lung. The scanning parameters were as follows: spiral scanning, a tube voltage of 120 keV, automatic tube current regulation, and a layer thickness of 5 mm. A bone algorithm was used for reconstruction, and the layer thickness after reconstruction was 1.25–2 mm. A total of 9876 HRCT images were obtained from 150 IIP patients, which were output in DICOM format and labeled in BMP format by two radiologists with 10 years of clinical experience. When the two doctors had differing opinions on the identification of an imaging feature, a third radiologist, who was senior to the two raters, judged the results. A total of 7 imaging features were marked, namely, ground glass opacities, reticulations, honeycombing, consolidations, emphysema, pleural effusion, and tractive bronchiectasis. The following labeling principles were followed: (1) selection of labeling area: only label abnormal imaging features displayed on single-slice CT; (2) labelling the extent of the imaging features: the lesion label can include <1 cm normal tissue, and diffusely distributed imaging features should include all lesions rather than only local changes; and (3) different imaging features shall be marked separately. If diffuse lesion contained multiple different imaging features, the features were marked separately, allowing a small amount of overlap.

### 2.3. Development of the Imaging Feature Recognition Model

The convolutional neural network (CNN)-based artificial intelligence platform was provided by OpenBayes (Tianjin) IT Co., Ltd. The backbone network of the model was U-net and was based on a full convolution network (FCN). This improved the accuracy without increasing the computational cost. The development of the model in this paper included three parts: chest CT image input, imaging feature extraction, and imaging feature recognition code output. Pyramidal convolution and a pooling module were used for feature extraction, including 4-layer downsampling and 4-layer upsampling. The feature map produced by the downsampling layers can avoid deep sampling and be spliced to the pair of maps at the upsampling end. The model then collects and integrates feature vectors of different sizes, performs multichannel feature extraction for vectors of different dimensions through multilayer convolution, and then implements machine learning to output the recognition codes for various imaging features. The information obtained from pyramidal pooling is more representative than that from global pooling. Through the U-net algorithm, we established a CNN and obtained the imaging feature recognition model.

### 2.4. Artificial Intelligence-Based Percentage Area Calculation for Different Imaging Features

Using the skimage library, lung tissue was identified by removing the parts of the images corresponding to hilar and mediastinal tissue. The number of pixels corresponding to the lung tissue and the different imaging features in each CT slice were then calculated, and the ratios of the latter to the former were used to define the percentage area of the different imaging features. The final percentage area for each patient was the sum of the percentages for all CT slices.

### 2.5. Establishment of a Glucocorticoid Sensitivity Prediction Model

Taking the proportions of different imaging features as the input and glucocorticoid sensitivity as the output, prediction models were established by using the k-nearest neighbor (KNN) algorithm and support vector machine (SVM) algorithm, and the advantages and disadvantages of the two algorithms were compared.

**Figure 2:**
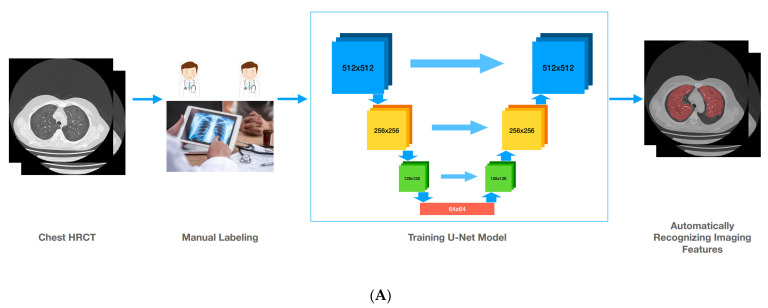

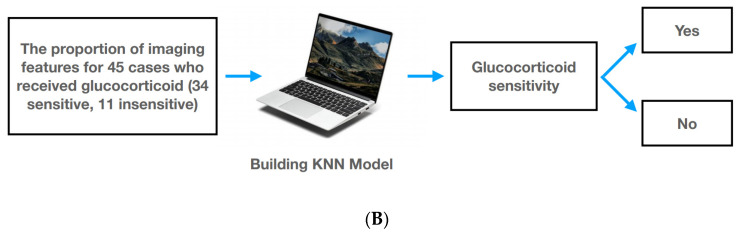
(**A**) The flowchart for developing a U-net model to automatically recognize imaging features. Chest HRCT, chest high-resolution computed tomography. Two radiologists labeled the chest HRCT of 150 IIP patients. Then, we developed a U-net model to recognize imaging features. When we placed one patient’s chest HRCT into the model, the model could recognize the different imaging features automatically. (**B**). The flowchart for developing a KNN model to automatically judge glucocorticoid sensitivity. KNN, k-nearest neighbor. The proportion of imaging features for 45 cases was calculated automatically. When we placed one patient’s chest HRCT into the artificial intelligence model which included the U-net model, automatic calculation, and the KNN model, it could firstly recognize imaging features and then calculate the proportion of imaging features, and finally judge the glucocorticoid sensitivity.

The entire flowchart for establishing an artificial intelligence-based model to assess the correlation between imaging features and glucocorticoid sensitivity is presented as Figure 2.

### 2.6. Assessment of the Correlation between Imaging Features and Glucocorticoid Sensitivity with Traditional Statistical Methods

First, we calculated the percentage area of the different imaging features through manually labeling and compared them in the glucocorticoid-sensitive group and the glucocorticoid-insensitive group. Next, we analyzed the correlation between glucocorticoid efficacy and the values that were statistically significant. Traditional statistical methods were implemented in IBM SPSS Statistics version 25 (IBM SPSS Inc., Chicago, IL, USA) software. In single-factor analysis, the normality test was conducted for measurement data; those that did not conform to a normal distribution are indicated as the median (minimum value~maximum value), and the Mann–Whitney U test was used for intergroup comparisons. Count data are indicated as n, and the chi-square test was used for intergroup comparisons. Unconditional logistic regression analysis was used for multivariate analysis. With glucocorticoid sensitivity as the dependent variable and the significant variables in the single-factor analysis as the independent variables, a logistic regression model was used for correlation analysis. A two-tailed *p* < 0.05 indicates statistical significance.

## 3. Results

Among the 150 IIP patients, there were 105 males and 45 females, and the average age was 65.95 (22–84) years. A total of 9876 HRCT slices were obtained, of which 7640 were labeled. The numbers of different imaging features manually labelled is shown in Table 1. Due to the limited imaging data on emphysema, pleural effusion, and tractive bronchiectasis, no artificial intelligence algorithm or model was established for identifying those conditions. For the remaining features, 3900 slices containing images of ground glass opacities, reticulations, honeycombing, and consolidations were used to train the AI model; 1250 were used for validation; and 120 were used to test the model.

**Figure 3:**
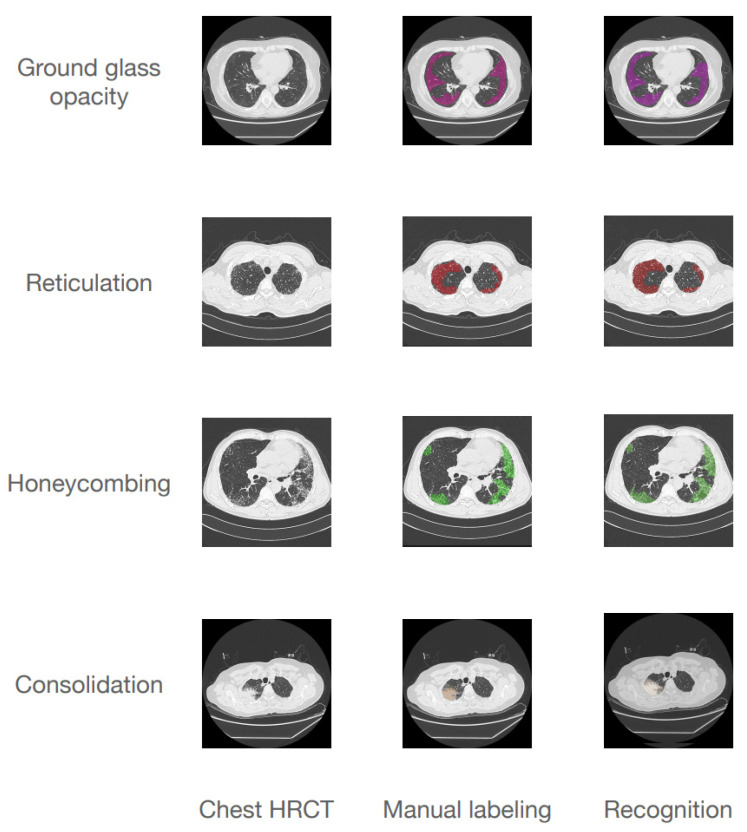
Results of the U-net model in identifying four imaging features in IIP patients. HRCT, high-resolution computed tomography. Chest HRCT line shows the four imaging features in HRCT. Manual labeling line shows the four imaging features labeled by radiologists. Recognition line shows the four imaging features recognized using the U-net model.

The overall accuracy and mean intersection over union (mIoU) value of the U-Net model in identifying the imaging features were 0.9755 and 0.4296, respectively. On an individual feature basis, the accuracies were as follows: ground glass opacity, 0.9691 (2050/2115); reticulation, 0.9669 (3240/3351); honeycombing, 0.9854 (860/873); and consolidation, 0.9919 (943/951). The mIoU values were as follows: ground glass opacity, 0.4307; reticulation, 0.3873; honeycombing, 0.3462; and consolidation, 0.1785. They are shown in Figure 3.

Taking the percentage area of the different imaging features as the input and glucocorticoid sensitivity as the output, artificial intelligence-based models were established using the KNN and SVM algorithms, and the accuracy of the two algorithms was compared, as shown in Table 2. The accuracy, macroaverage, and weighted average of the KNN algorithm were better than those of the SVM algorithm, which shows that the KNN algorithm can better determine a patient’s glucocorticoid sensitivity.

The traditional Mann–Whitney U test and chi-square test were used to compare the proportions of imaging features between the glucocorticoid-sensitive group and the glucocorticoid-insensitive group. To obtain more meaningful results, unconditional logistic regression was used to analyze the correlation between the imaging features and glucocorticoid efficacy. There were 34 patients in the glucocorticoid-sensitive group, which included 15 males with an average age of 62 years, and 11 patients in the glucocorticoid-insensitive group, including 6 males with an average age of 61 years. There was no significant difference in age or sex between the two groups. A comparison of the results between the groups showed that the glucocorticoid-sensitive group had a higher percentage area of ground glass opacities and consolidations, while the glucose-insensitive group had a higher percentage area of reticulations and honeycombing; the differences were statistically significant (Table 3). Logistic regression analysis was performed using the percentage area of ground glass opacities, the combination of reticulations and honeycombing, and consolidations as independent (continuous) variables and glucocorticoid sensitivity as the dependent variable, and the results are shown in Table 4. The findings indicate that the combination of reticulations and honeycombing in HRCT was negatively correlated with glucocorticoid sensitivity. In other words, the greater the total percentage area of reticulations and honeycombing, the worse the glucocorticoid sensitivity was for the patient.

## 4. Discussion

In our study, 45 patients were treated with glucocorticoids. Using traditional statistical methods, the total percentage area of reticulations and honeycombing in the glucocorticoid-insensitive group were greater than that in the glucocorticoid-sensitive group and was negatively correlated with glucocorticoid efficacy. In other words, a greater number of reticulations and honeycombing was associated with less sensitivity to glucocorticoids, which corresponds to previous research results.

Advancements in artificial intelligence technology have allowed its widespread use in the imaging identification of lung diseases, including automatic identification, size measurement, malignant nodule recognition in lung CT, the diagnosis and prognosis of COVID-19 pneumonia and tuberculosis, and drug resistance assessment. CNNs are a widely used form of artificial intelligence for imaging recognition. By simulating the activity of human neurons, the CNN takes the image features at the input end and extracts them through the processes of the convolution layers, pooling layers, and fully connected layers. The convolution and pooling layers extract the imaging features and combine them over multiple cycles, performing repeated learning and ultimately outputting the imaging features. In principle, a large amount of data is needed for training and then machine learning; therefore, for common diseases, a highly accurate recognition model can be created by obtaining a large amount of clinical and imaging data. The incidence of interstitial lung disease (ILD) is low; however, limited clinical data are available for training. Similarly, few studies have been conducted on CNNs in interstitial lung disease to date. Walsh et al. [14] applied a CNN to train a model on the HRCT images of 929 patients with interstitial lung disease, validated it with the data from 89 patients, and tested it with the data from 139 patients. The output of the model was usual interstitial pneumonia (UIP), non-UIP, or possible UIP; it achieved an accuracy of 0.764, a sensitivity of 0.793, a specificity of 0.901, and a C index of 0.85, which is equivalent to the accuracy of general radiologists. The time that the CNN model needed to identify UIP was substantially reduced relative to humans, assessing the HRCT images of 150 patients in only 2.31 s, indicating its potential usefulness in screening centers. However, only four slices of the lung CT images are selected and combined at the input stage, and the output is the IPF diagnosis and the presence of UIP, while specific imaging features are not identified. Anthimopoulos et al. [15] trained a model on the chest CT images of 120 patients with IIP to recognize six features, namely, ground glass opacities, reticulations, honeycombing, consolidations, micronodules, and normal lung tissue, and achieved an accuracy of 0.8561. They applied this training set to four other CNN algorithms including AlexNet, a pretrained AlexNet (AlexNetP), and VGG-Net, but their areas under the curve (AUCs) were less than that of the first model. However, ordinary chest CT was used in this study, and its resolution is worse than that of HRCT typically used for ILD imaging feature recognition. Huang et al. [16] designed a new CNN to classify five ILD features, namely, normal tissue, ground glass opacities, emphysema, micronodules, and fibrosis. A new two-stage transfer learning (TSTL) method was proposed to address the lack of training data. This method uses the knowledge learned from sufficient source texture data and auxiliary, unlabeled lung CT data to reach the target domain and an unsupervised method to learn unlabeled data to optimize the objective function, composed of prediction confidence and mutual information. The results showed that this CNN structure achieves ideal performance superior to that of the majority of most advanced structures. Further comparative analysis showed that the proposed TSTL strategy had good feasibility and certain advantages over existing transfer learning strategies. Christodoulidis et al. [17] and Gao et al. [18] divided original, standard-resolution CT images into three levels according to the attenuation of the HU value; took the attenuation of the three levels as the input; and applied the migration learning method to train the network to distinguish ILD imaging features.

In this study, a U-Net and KNN algorithm was used to establish a model for assessing glucocorticoid efficacy in IIP patients. The whole process of the model was divided into two parts. In the first part, U-Net was used to create HRCT recognition models for ground glass opacities, reticulations, honeycombing, and consolidations. The overall recognition accuracy was 0.9755, suggesting that the above four imaging features can be accurately judged by the model by inputting HRCT images. On an individual imaging feature basis, the accuracy for ground glass opacities was the highest, which is related to the large number of manually labelled images with ground glass opacities. The total mIoU value for the imaging features in this study was 0.4296; again, the mIoU value for ground glass opacities was the highest, while that of consolidations was the lowest, suggesting that the segmentation accuracy for imaging features of the artificial intelligence model was not ideal; this may be related to the small proportion of abnormal imaging features in the training set and the low discriminability between the identified imaging features and the surrounding lung tissue. The model performance can be improved by increasing the amount of data in the training set and the number of patients with high proportions of abnormal image feature areas. The U-Net used in this paper adopts pyramidal convolution and pooling layers and applies upsampling and downsampling methods, resulting in the extraction of more comprehensive information with fewer data sets and increasing the accuracy of the output results.

**Figure 4:**
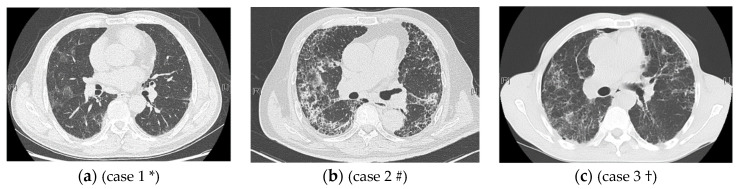
Different imaging manifestations of HRCT may affect the judgment of AI on glucocorticoid sensitivity. * Case 1 HRCT mainly shows ground glass opacity and was correctly identified as sensitive to glucocorticoids using the AI model. # Case 2 HRCT shows mainly reticulations and honeycombing and was correctly identified as insensitive to glucocorticoids using the AI model. † Case 3 is mainly characterized by ground glass opacities, reticulations, and honeycombing and was incorrectly identified as sensitive to glucocorticoids using the AI model.

In the second part of the artificial intelligence method of this study, after identifying the imaging features with the CNN model and calculating the area percentages of each imaging feature, the latter were used as the input and glucocorticoid sensitivity as the output to develop correlation models with the KNN and SVM algorithms. Since only 45 patients were treated with glucocorticoids, the training set was used as the validation set, and the KNN and SVM algorithms were applied to assess the correlation of the imaging findings with glucocorticoid sensitivity, achieving accuracies of 0.82 and 0.80, respectively. The KNN algorithm slightly outperformed the SVM algorithm, suggesting that the KNN model had greater resolution in identifying glucocorticoid sensitivity. Thirty-four patients were included in the glucocorticoid-sensitive group, and eleven patients were included in the glucocorticoid-insensitive group. The accuracy of the model in identifying glucocorticoid sensitivity was 1, while the positive predictive value of the model in identifying glucocorticoid insensitivity was only 0.27. The reasons for the low positive rate of the model in identifying patients with glucocorticoid insensitivity are as follows: The algorithm design does not attempt to identify glucocorticoid sensitivity by factors other than the area percentages of the imaging features. In addition to previous research results indicating that the combination of reticulations and honeycombing areas on HRCT is related to glucocorticoid sensitivity, different HRCT imaging features may be present, and their different relative area percentages could result in different glucocorticoid effects. By reviewing the HRCT images of all patients treated with glucocorticoids in this study, we observed that IIP patients typically presented with one of the following patterns: mainly ground glass opacities with almost no reticulations or honeycombing; mainly reticulations or honeycombing but almost no ground glass opacities; and ground glass opacities, reticulations, honeycombing, and consolidations coexisting in the same or similar proportions. When the imaging features were primarily ground glass and there were few reticulations and little honeycombing, the patient was more likely to be sensitive to glucocorticoids, and the accuracy of the AI model was high. When the imaging features were ground glass opacities, reticulations, and honeycombing, the model had to consider the ratio of the area of ground glass opacities to that of the combination of reticulations and honeycombing; a greater area ratio was more predictive of glucocorticoid sensitivity. If the imaging features included ground glass opacities, reticulations, honeycombing, and consolidations, the greater the ratio of the area of ground glass opacities plus consolidations to the area of reticulations plus honeycombing, the more sensitive the patient was likely to be to glucocorticoids. Figure 4a shows the chest CT of a 63-year-old male, which primarily presents with ground glass opacity (accounting for 21.6% of the area), while reticulations account for 0.06%. The AI model labeled this patient as sensitive to glucocorticoids, matching the actual glucocorticoid effect. A 67-year-old male, whose CT images are shown in Figure 4b, was correctly identified as insensitive to glucocorticoids through the AI model. Reticulations accounted for 49.8% of the area, honeycombing accounted for 15.3%, and the sum of the two accounted for 65.1%. The CT images of a 64-year-old male shown in Figure 4c can be mainly characterized by ground glass opacities (15.1% of the area), reticulations (37.54%), and honeycombing. The AI model classified this patient as glucocorticoid sensitive, but he was actually insensitive to glucocorticoids. Overall, among the samples of this study, the accuracy in identifying the glucocorticoid-sensitive group was acceptable, while that in identifying glucocorticoid-insensitive individuals needs to be improved. By expanding the number of patients, other factors that may affect the efficacy of glucocorticoids can be added to construct the AI model.

Our study has some limitations. First, given the retrospective nature of the study, there was likely some selection bias, which can be addressed with a multicenter, prospective study. Second, the HRCT images were derived from two CT machines in our hospital; the lack of imaging data from other types of CT machines can lead to overfitting and inefficient generalization when creating the artificial intelligence model. Future studies could address this by adding data from a variety of CT machines in other research centers. Third, the number of patients treated with glucocorticoids was small, hindering the generalizability of the artificial intelligence model. This should be increased in future research. Fourth, the manually labeled imaging features were only submitted to the senior professional doctor for review when there were differences between the two labelling radiologists. This could have resulted in labeling errors for similar imaging features, which can be improved by increasing the number of labelling radiologists and the number of image features labeled by the senior professional doctor.

## 5. Conclusions

This study created an artificial intelligence model to recognize the imaging features of HRCT and preliminarily established a model for identifying the correlation between imaging features and glucocorticoid sensitivity in patients with IIP.

## Figures and Tables

**Table 1:** Number of manually labeled imaging features.

Imaging Feature	Number Manually Labeled
ground-glass opacity	2115
reticulation	3351
honeycombing	873
consolidation	951
emphysema	221
pleural effusion	38
tractive bronchiectasis	91

**Table 2:** Results of the glucocorticoid sensitivity models established using KNN and SVM algorithms.

Algorithms	Indexes	Accuracy	Recall Rate	F1-Score	Cases
KNN	Glucocorticoid sensitivity	0.81	1	0.89	34
	Glucocorticoid insensitivity	1	0.27	0.43	11
	Average accuracy			0.82	45
	Macroaverage	0.9	0.64	0.66	45
	Weighted mean	0.86	0.82	0.78	45
SVM	Glucocorticoid sensitivity	0.79	1	0.88	34
	Glucocorticoid insensitivity	1	0.18	0.31	11
	Average accuracy			0.8	45
	Macroaverage	0.9	0.59	0.6	45
	Weighted mean	0.84	0.8	0.74	45

**Table 3:** Imaging features of the glucocorticoid-sensitive group and glucocorticoid-insensitive group.

Variables	Glucocorticoid-Sensitive Group (*n* = 34)	Glucocorticoid-Insensitive Group (*n* = 11)	Statistics	*p* Value
Proportion of ground glass opacity (%)	17.45 (0, 77.62)	6.70 (0, 64.11)	−2.970 †	0.002
Proportion of reticulation (%)	7.04 (0, 47.61)	26.19 (0, 49.82)	−3.342 †	0.001
Proportion of honeycombing (%)	0.37 (0, 12.34)	8.77 (0, 39.51)	−3.725 †	0.012
Proportion of the combination of reticulations and honeycombing (%)	7.42 (0, 47.90)	34.96 (0, 65.10)	−3.636 †	0.000
Number of patients for whom the proportion of the combination of reticulations and honeycombing ≥20%	6 (17.64)	9 (81.81)	15.400 *	0.000
Proportion of consolidations (%)	2.16 (0, 12.76)	0.014 (0, 0.1)	−2.582 †	0.015

Data are expressed as *n* (%) or the median (interpercentile range). * Chi-square test; † Mann–Whitney U test.

**Table 4:** Multivariate logistic regression analysis was used to determine the correlation between imaging features and glucocorticoid sensitivity.

Variables	ß	S.E.	OR (95% CI)	*p* Value
Ground glass opacities	0.012	0.030	1.013 (0.954, 1.074)	0.679
Combination of reticulations and honeycombing	0.113	0.044	1.120 (1.027, 1.221)	0.010
Consolidations	−22.887	13.811	0.000 (0.000, 65.480)	0.097

ß, regression coefficient; S.E., standard error; OR, odds ratio.

## Data Availability

The data presented in this study are available on reasonable request from the authors (Ling Jiang and Bei He).

## References

[1] Wu X.Y., Yi X.H., Li H.P., Luo B.F., Li X., Wang Y.L., Zeng Y., Rui W.W., Zhu X.Y., Li X.J. (2010). A study on the efficacy of glucocorticoid therapy for idiopathic nonspecific interstitial pneumonia. Chin. J. Tuberc. Respir. Dis..

[2] Chen Y.H., Yao W.Z., Yang W., Zhao M.W. (2002). Study of 31 patients with idiopathic interstitial pneumonia treated with glucocorticosteroid. J. Peking. Univ. (Health Sci.).

[3] Yang W., He B., Shan Y. (2009). High-resolution CT semi-quantitative score may have a role in predicting short-term response to corticosteroid therapy in idiopathic interstitial pneumonias. Chin. J. Tuberc. Respir. Dis..

[4] Gay S.E., Kazerooni E.A., Toews G.B., Lynch J.P., Gross B.H., Cascade P.N., Spizarny D.L., Flint A., Schork M.A., Whyte R.I. (1998). Idiopathic pulmonary fibrosis: Predicting response to therapy and survival. Am. J. Respir. Crit Care Med..

[5] Quigley M., Hansell D.M., Nicholson A.G. (2006). Interstitial lung disease--the new synergy between radiology and pathology. Histopathology.

[6] Hunninghake G.W., Lynch D.A., Galvin J.R., Gross B.H., Müller N., Schwartz D.A., King T.E., Lynch J.P., Hegele R., Waldron J. (2003). Radiologic findings are strongly associated with a pathologic diagnosis of usual interstitial pneumonia. Chest.

[7] Mura M., Ferretti A., Ferro O., Zompatori M., Cavalli A., Schiavina M., Fabbri M. (2006). Functional predictors of exertional dyspnea, 6-min walking distance and HRCT fibrosis score in idiopathic pulmonary fibrosis. Respiration.

[8] Setio A.A., Ciompi F., Litjens G., Gerke P., Jacobs C., van Riel S.J., Winkler Wille M.M., Naqibullah M., Sanchez C.I., van Ginneken B. (2016). Pulmonary nodule detection in CT images: False positive reduction using multi-view convolutional networks. IEEE Trans. Med. Imaging.

[9] Dou Q., Chen H., Yu L., Qin J., Heng P.A. (2017). Multilevel contextual 3-D CNNs for false positive reduction in pulmonary nodule detection. IEEE Trans. Biomed. Eng..

[10] Li L., Qin L., Xu Z., Yin Y., Wang X., Kong B., Bai J., Lu Y., Fang Z., Song Q. (2020). Using artificial intelligence to detect COVID-19 and community-acquired pneumonia based on pulmonary CT: Evaluation of the diagnostic accuracy. Radiology.

[11] Wang S., Kang B., Ma J., Zeng X., Xiao M., Guo J., Cai M., Yang J., Li Y., Meng X. (2021). A deep learning algorithm using CT images to screen for Corona virus disease (COVID-19). Eur. Radiol..

[12] Chen H., Guan C.S., Yan S., Chen Q.Y., Zhang Y.J., Chen B.D., Xie R.M. (2021). Application of artificial intelligence in early imaging diagnosis of COVID-19. J. Pract. Radiol..

[13] Gao X.W., Qian Y. (2018). Prediction of multidrug-resistant TB from CT pulmonary images based on deep learning techniques. Mol. Pharm..

[14] Walsh S.L.F., Calandriello L., Silva M., Sverzellati N. (2018). Deep learning for classifying fibrotic lung disease on high-resolution computed tomography: A case-cohort study. Lancet. Respir. Med..

[15] Anthimopoulos M., Christodoulidis S., Ebner L., Christe A., Mougiakakou S. (2016). Lung pattern classification for interstitial lung diseases using a deep convolutional neural network. IEEE Trans. Med. Imaging.

[16] Huang S., Lee F., Miao R., Si Q., Lu C., Chen Q. (2020). A deep convolutional neural network architecture for interstitial lung disease pattern classification. Med. Biol. Eng. Comput..

[17] Christodoulidis S., Anthimopoulos M., Ebner L., Christe A., Mougiakakou S. (2017). Multisource transfer learning with convolutional neural networks for lung pattern analysis. IEEE J. Biomed. Health Inform..

[18] Gao M., Bagci U., Lu L., Wu A., Buty M., Shin H.C., Roth H., Papadakis G.Z., Depeursinge A., Summers R.M. (2018). Holistic classification of CT attenuation patterns for interstitial lung diseases via deep convolutional neural networks. Comput. Methods Biomech. Biomed. Eng. Imaging Vis..

